# Expression of *HK2, PKM2,* and *PFKM* Is Associated with Metastasis and Late Disease Onset in Breast Cancer Patients

**DOI:** 10.3390/genes13030549

**Published:** 2022-03-20

**Authors:** Mehreen Ishfaq, Nabiha Bashir, Syeda Kiran Riaz, Shumaila Manzoor, Jahangir Sarwar Khan, Yamin Bibi, Rokayya Sami, Amani H. Aljahani, Saif A. Alharthy, Ramla Shahid

**Affiliations:** 1 Department of Biosciences, COMSATS University Islamabad, Islamabad 44000, Pakistan; mehreenishfaq@gmail.com (M.I.); nbsiddique56@gmail.com (N.B.); 2 Department of Molecular Biology, Shaheed Zulfiqar Ali Bhutto Medical University, Islamabad 44000, Pakistan; syedakiranriaz@szabmu.edu.pk; 3 National Veterinary Lab, National Agricultural Research Centre, Islamabad 44000, Pakistan; smnvl786@gmail.com; 4 Department of General Surgery, Rawalpindi Medical University, Rawalpindi 46000, Pakistan; jskdr@hotmail.com; 5 Department of Botany, PMAS-Arid Agriculture University Rawalpindi, Rawalpindi 46300, Pakistan; dryaminbibi@uaar.edu.pk; 6 Department of Food Science and Nutrition, College of Sciences, Taif University, P.O. Box 11099, Taif 21944, Saudi Arabia; rokayya.d@tu.edu.sa; 7 Department of Physical Sport Science, College of Education, Princess Nourah bint Abdulrahman University, P.O. Box 84428, Riyadh 11671, Saudi Arabia; ahaljahani@pnu.edu.sa; 8 Department of Medical Laboratory Sciences, Faculty of Applied Medical Sciences, King Abdulaziz University, P.O. Box 80216, Jeddah 21589, Saudi Arabia; saalharthy@kau.edu.sa; 9 King Fahd Medical Research Center, King Abdulaziz University, P.O. Box 80216, Jeddah 21589, Saudi Arabia

**Keywords:** aerobic glycolysis, breast cancer, *HK2*, *PFKM*, *PKM2*, Warburg effect

## Abstract

The reprogramming of energy metabolism is one of the hallmarks of cancer and is crucial for tumor progression. Altered aerobic glycolysis is a well-known characteristic of cancer cell metabolism. In the present study, the expression profiles of key metabolic genes (*HK2*, *PFKM,* and *PKM2*) were assessed in the breast cancer cohort of Pakistan using quantitative polymerase chain reaction (qPCR) and IHC. Expression patterns were correlated with molecular subtypes and clinical parameters in the patients. A significant upregulation of key glycolytic genes was observed in tumor samples in comparison to their adjacent controls (*p* < 0.0001). The expression of the studied glycolytic genes was significantly increased in late clinical stages, positive nodal involvement, and distant metastasis (*p* < 0.05). *HK2* and *PKM2* were found to be upregulated in luminal B, whereas *PFKM* was overexpressed in the luminal A subtype of breast cancer. The genes were positively correlated with the proliferation marker *Ki67* (*p* < 0.001). Moreover, moderate positive linear correlations between *HK2* and *PKM2* (r = 0.476), *HK2* and *PFKM* (r = 0.473), and *PKM2* and *PFKM* (r = 0.501) were also observed (*p* < 0.01). These findings validate that the key regulatory genes in glycolysis can serve as potential biomarkers and/or molecular targets for breast cancer management. However, the clinical significance of these molecules needs to be further validated through in vitro and in vivo experiments.

## 1. Introduction

Metabolic reprogramming or disturbance in energy metabolism is the most common characteristic in malignant tumors and one of the hallmarks of cancer [1]. To maintain rapid growth, cancer cells can alter their capability to metabolize lipids, carbohydrates, and proteins [2]. Normal cells follow oxidative phosphorylation (OXPHOS) by consuming glucose and oxygen in order to produce energy and shift metabolism to glycolysis in hypoxic conditions (oxygen-deprived environments) to fulfill their energy needs [3]. On the contrary, cancer cells mainly produce energy by glycolysis, even in the abundance of oxygen, which has been termed the “Warburg effect” [4].

Cancer glycolysis is a key step in oncogenic activation and tumor progression. Glycolysis leads to a breakdown of glucose molecules to pyruvate, with the help of hexokinase (HK), phosphofructokinase (PFK), and pyruvate kinase (PK) [5]. These enzymes exist in various isoforms, which are encoded by their specific genes. To date, four isoforms of HKs have been characterized in mammals [6]. Of these, the one encoded by the *HK2* (hexokinase 2) gene was found to be overexpressed in a variety of cancers, i.e., colorectal [7], lung [8], digestive [9], and liver cancers [10]. *HK2* catalyzes the initial step during glycolysis by phosphorylating glucose to produce glucose-6-phosphate (G-6P). Moreover, knockdown of *HK2* leads to tumor growth inhibition in prostate, glioblastoma, and pancreatic cancers [8]. In addition, an association of hyperactive glycolysis with *HK2* overexpression was found in hepatocellular carcinomas [11]. An increase in overall survival and decreased tumor burden was seen upon *HK2* deletion in KRAS-driven lung cancer and ERBB2 driven breast cancer [8].

Phosphofructokinase is the key rate-limiting enzyme of all the glycolysis regulatory catalytic complexes. It is responsible for controlling the maximum percentage of glycolytic activity [12]. Conversion of fructose-6-phosphate to fructose-1,6-bisphosphate in glycolysis is mediated by phosphofructokinase with a release of energy [13]. Out of the three isoforms, the PFKM is a crucial regulatory target encoded by the *PFKM* (phosphofructokinase muscle) gene, as it serves as an activator of muscle glycolysis, which is critical for cancer dissemination [14]. Moreover, an in silico study reported *PFKM* as a potential therapeutic target for cancer and aerobic glycolysis. *PFKM* genetic mutation associated with different cancers, including human melanomas, breast cancer, bladder cancer, non-small-cell lung cancer, and glioma has also been observed [15].

*PKM2* (pyruvate kinase isozyme M2) plays a key role in the regulation of cell metabolism by catalyzing the final step of glycolysis [16]. It converts phosphoenolpyruvate to produce pyruvate and ATP [17]. Studies have shown that *PKM2* gene expression is critical for aerobic glycolysis in cancer cells [18]. *PKM2* also functions as a coactivator of HIF1 (hypoxia-inducible factor) to promote Warburg metabolism [19]. It has been reported that a shift in metabolism from glycolysis to OXPHOS occurred when mice were engineered to express *PKM1* instead of *PKM2* [20]. The role of *PKM2* has been studied in a variety of cancers including melanoma, lung, cervical, and colorectal cancers [21].

Dysregulation of key metabolic genes, namely, *HK2*, *PFKM,* and *PKM2*, has the potential to disrupt glycolytic metabolism and remove additional barriers against tumor progression [22,23]. Hence, the current study aims to explore the expression profiles of these genes in the breast cancer cohort of Pakistan. In addition, the correlation of glycolytic markers with clinicopathological parameters, the *Ki67* proliferation marker, and molecular subtypes in breast cancer was also assessed. This study will provide insight into the potential role of glycolytic genes in breast tumor progression and metastasis.

## 2. Materials and Methods

### 2.1. Inclusion and Exclusion Criteria

Patients who were diagnosed with breast cancer were included in the study. Patients with a history of hereditary/familial diseases, hepatitis, and HIV (human immunodeficiency virus) were excluded.

### 2.2. Tissue and Data Collection

The current study was conducted after formal approval from biosafety and bio-ethical committees of COMSATS University and affiliated hospitals. The study cohort consisted of freshly excised breast tumor specimens along with their adjacent normal (2 cm away from the site of the tumor) tissues (*n* = 120; tumor tissue = 60; control tissue = 60). The samples were obtained with informed patient consent. Demographic data, including age, menopausal status, laterality, and clinicopathological findings (including tumor grade, stage, size, nodal involvement, and receptor status), were also retrieved. Samples were immediately transferred to RNA later solution and stored at −80 °C until further use.

### 2.3. RNA Extraction and cDNA Synthesis

Samples from both tumor tissues and adjacent controls were homogenized for RNA isolation using TRIzol^®^ Reagent. The purity of RNA was measured using a spectrophotometer at absorbances of 260 nm and 280 nm. cDNA (complementary DNA) was generated from 0.5 µg of RNA using Revert Aid First Strand cDNA Synthesis Kit (Thermo Scientific, San Diego, USA) according to manufacturer’s instructions.

### 2.4. Primer Designing

Primers were designed using an online available tool PRIMER 3. Primer specificity was confirmed using NCBI primer Blast. Primer sequences for the studied genes, i.e., *HK2*, *PKM2*, *PFKM*, *Ki67,* and *β-actin,* along with their product sizes, are listed in Table 1.

### 2.5. Quantitative Real Time PCR

The resulting cDNA was then subjected to qPCR using SYBR green qPCR Master Mix (Thermo Scientific, San Diego, USA). The qPCR reaction was performed with initial denaturation for 12 min at 95 °C followed by 35 cycles of amplification (denaturation for 45 s at 94 °C, annealing at 59 °C for 30 s, and elongation for 30 s at 72 °C) as per the manufacturer’s instructions using the Applied Biosystems 9200 system. *Β-actin* was used as an internal control.

### 2.6. Immuno-Histochemistry (IHC)

IHC was performed with these antibodies (200 µg/mL), anti-hexokinase II (HXK II (B-8); sc-374091; dilution 1:400; Santa cruz, CA, USA); anti-phosphofructokinase (PFK1 (E-4); sc-377346; dilution 1:500; Santa cruz, CA, USA); and anti-pyruvate kinase muscle (PKM (C-11); sc-365684; dilution 1:200; Santa cruz, CA, USA), using the methodology described earlier [24]. Protein expression of the respective genes was compared between adjacent normal tissues as controls and tumor samples retrieved from paraffin-embedded formalin-fixed blocks of breast cancer-affected patients.

### 2.7. Expression of Glycolytic Genes in TCGA Cohort

The GEPIA platform was used for the TCGA dataset to perform tumor vs. normal comparisons. Expressions of *HK2*, *PFKM*, and *PKM* transcripts in the breast cancer cohort (BRCA) were evaluated in comparison to normal breast tissue in TCGA. Student’s t-test and cut-off (*p* < 0.05) were used in the GEPIA platform. BRCA was comprised of 1085 tumors and 291 normal breast tissue samples.

### 2.8. Statistical Evaluation of Data

Livak’s method (2^−∆∆Ct^) [25] was used to measure the comparative mRNA expression of target genes (*HK2*, *PKM2*, and *PFKM*) in tumor samples compared to their respective adjacent controls. The results are expressed as mean ± SEM. Wilcoxon signed-rank test was performed for statistical comparison between tumors and controls. Non-parametric methods of statistical testing, including Kruskal-Wallis and Mann-Whitney U tests, were used to investigate the association of clinicopathological characteristics with *HK2*, *PKM2,* and *PFKM* expressions. Data for molecular subtypes of breast cancer were analyzed using one-way ANOVA. Spearman rank test was used to assess the correlation between the expression of the glycolytic genes and *Ki67*. A Kaplan–Meier curve was generated, and overall survival was analyzed using a log-rank (Mantel–Cox) test. All statistical analyses were performed using GraphPad Prism version 10 (GraphPad, La Jolla, CA, USA). The results were considered as significant at *p* < 0.05.

## 3. Results

### 3.1. Association of Glycolytic Gene Expression with Demographic Characteristics

**Figure 1:**
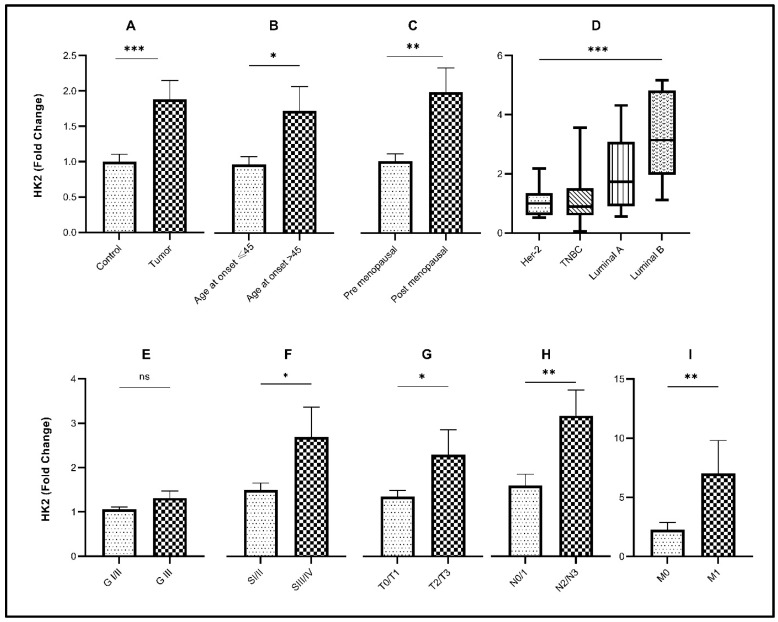
Association of *HK2* gene expression with various clinicopathological parameters and molecular subtypes. Fold change of *HK2* gene in (**A**) control vs. tumor tissues; (**B**) different age groups of disease onset; (**C**) menopausal status; (**D**) molecular subtypes of breast cancer; (**E**) tumor grade; (**F**) tumor stage; (**G**) tumor size; (**H**) nodal involvement; (**I**) metastasis. Significance level * *p* < 0.05, ** *p* < 0.001, *** *p* < 0.0001.

**Figure 2:**
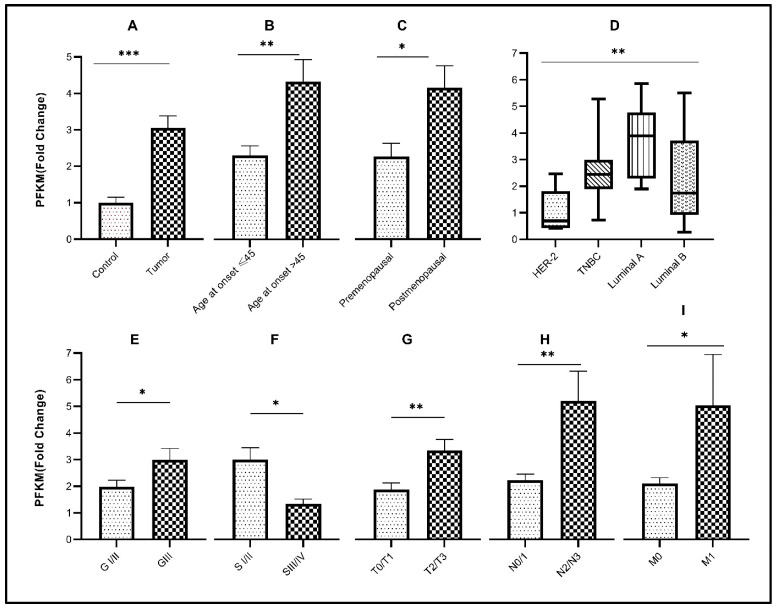
Association of *PFKM* gene expression with various clinicopathological parameters and molecular subtypes. Fold change of *PFKM* gene in (**A**) control vs. tumor tissues; (**B**) different age groups of disease onset; (**C**) menopausal status; (**D**) molecular subtypes of breast cancer; (**E**) tumor grade; (**F**) tumor stage; (**G**) tumor size; (**H**) nodal involvement; (**I**) metastasis. Significance level * *p* < 0.05, ** *p* < 0.001, *** *p* < 0.0001.

**Figure 3:**
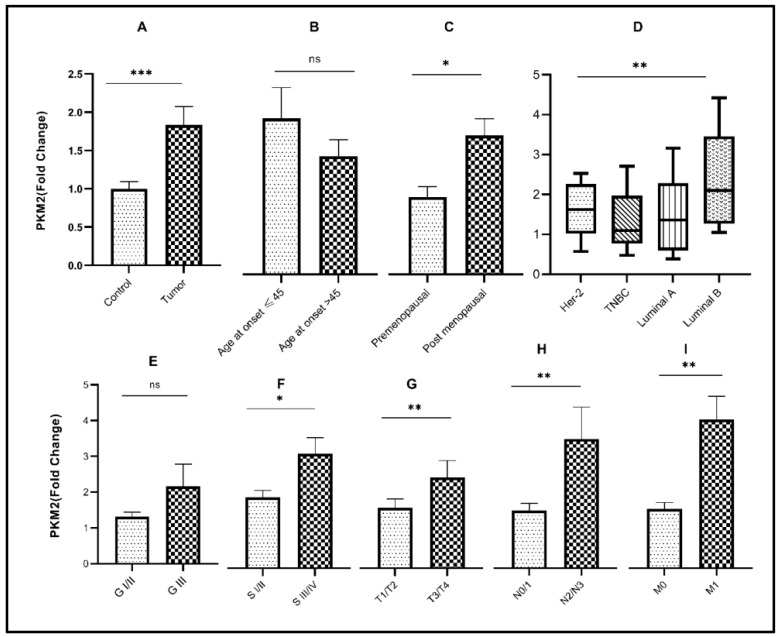
Association of *PKM2* gene expression with various clinicopathological parameters and molecular subtypes. Fold change of *PKM2* gene in (**A**) control vs. tumor tissues; (**B**) different age groups of disease onset; (**C**) menopausal status; (**D**) molecular subtypes of breast cancer; (**E**) tumor grade; (**F**) tumor stage; (**G**) tumor size; (**H**) nodal involvement; (**I**) metastasis. Significance level * *p* < 0.05, ** *p* < 0.001, *** *p* < 0.0001.

The mean age of breast cancer patients included in this study was 45 years with an age range between 24 and 70 years. In the cohort, 50% of patients were younger than 45 years at the time of diagnosis. Transcript levels of *HK2* (*p*< 0.05) and *PFKM* (*p* < 0.001) were significantly elevated in older patients (age > 45 years) compared to younger patients. Expression of the glycolytic genes with respect to age at disease diagnosis is also indicated in Table 2 and Table 3, and Figure 1B, Figure 2B, and Figure 3B. Accordingly, significant overexpression of *HK2, PKM2,* and *PFKM* genes (*p* < 0.05) was observed in postmenopausal women in comparison to premenopausal women (Table 3 and Figure 1C, Figure 2C, and Figure 3C).

### 3.2. Relative Expression of Glycolytic Genes in Breast Cancer Study Cohort

**Figure 4:**
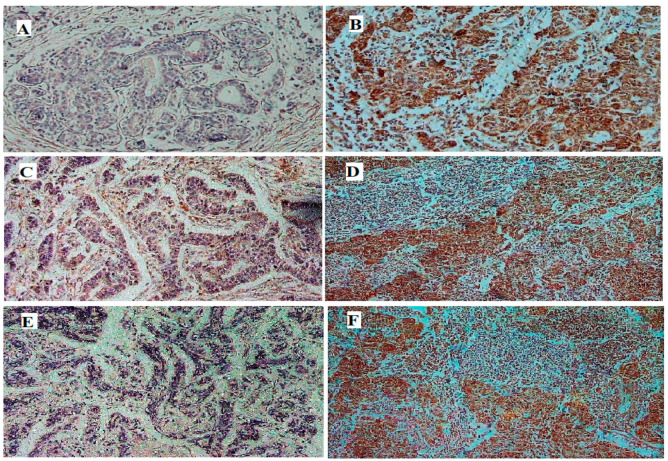
Immunostaining of representative breast tumor specimen compared with normal breast tissue. Protein expressions of *HK2* (**A**,**B**), *PFKM* (**C**,**D**), and *PKM* (**E**,**F**) were found to be higher in the tumor tissues (**B**,**D**,**F**) in comparison to adjacent normal tissues (**A**,**C**,**E**) (scale: 600 µm).

**Figure 5:**
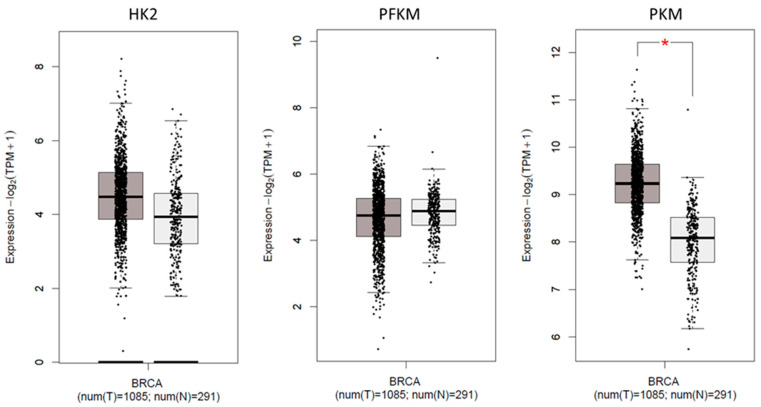
Expression of glycolytic markers in BRCA cohort from TCGA (* *p* < 0.05).

Significant upregulation (*p* < 0.0001) of the glycolytic genes (*HK2, PKM2*, and *PFKM*) was observed in breast tumor tissues in comparison to their respective controls (Table 3 and Figure 1A, Figure 2A, and Figure 3A). About 68% of tumors showed high expression of *HK2*, whereas 73% were high for *PKM2*. Interestingly, 80% of the tumors had overexpressed the *PFKM* gene as indicated in Table 2. Protein expressions of HK2, PFKM and PKM were found to be high in tumor tissues with cytoplasmic localization (Figure 4). Analysis from the BRCA dataset of TCGA also validated that the *PKM2* gene (*p* < 0.05) was significantly altered between tumors and normal breast samples. *HK2* expression was elevated in TCGA breast tumors as well. However, *PFKM* was not altered between tumors and normal breast tissue as shown in Figure 5.

### 3.3. Association of Glycolytic Genes with Clinicopathological Characteristics of the Study Cohort

Out of the three glycolytic genes, *PFKM* (*p* < 0.05) showed significant upregulation in higher-grade tumors as compared to low-grade tumors as indicated in Table 3 and Figure 2E. Significant overexpression of the glycolytic genes *HK2* and *PKM2* (*p* < 0.05) was observed in advanced clinical stages (stage III/IV) of breast cancer as compared to early stages (stage I/II), as shown in Figure 1F and Figure 3F. Comparably, significant overexpression of the *PFKM* gene (*p* < 0.05) was seen in the early clinical stages (SI/II) of breast cancer. Tumor stage, nodal involvement, and metastasis data were also retrieved for the given breast cancer cohort. A significant increase in the expression of these glycolytic genes was observed in increased tumor size, nodal metastasis, and distant metastasis (*p* < 0.05). Glycolytic gene associations due to tumor stage, nodal involvement, and metastasis are also shown in Table 3 and Figure 1G–I, Figure 2G–I, and Figure 3G–I.

For the current study cohort, follow-up data related to the overall survival of patients were obtained for a period of 36 to 48 months post-surgery. A Kaplan–Meier plot for the three glycolytic genes was generated based on the log-rank test. Kaplan–Meier graphs showed that the elevated expressions of *HK2* (HR = 1.95) and *PFKM* (HR = 2.03) are associated with poor prognosis in patients, as shown in Supplementary Figure S2.

### 3.4. Association of Glycolytic Genes with Molecular Subtypes of Breast Cancer

The present cohort consisted of 10% HER2, 21.7% TNBC, 21.7% luminal-A, and 46.6% luminal-B tumors. Among molecular subtypes, luminal-B had the highest expression of *HK2* and *PKM2* (*p* < 0.05), while *PFKM* showed the highest expression in the luminal A subtype (*p* < 0.05) as indicated in Figure 1D, Figure 2D, and Figure 3D. For each of the four subtypes, the expression of glycolytic genes *HK2, PFKM,* and *PKM2* was upregulated in tumors as compared to their paired control tissues (Supplementary Figure S1).

### 3.5. Correlation between Glycolytic Genes and Ki67 at mRNA Level

**Figure 6:**
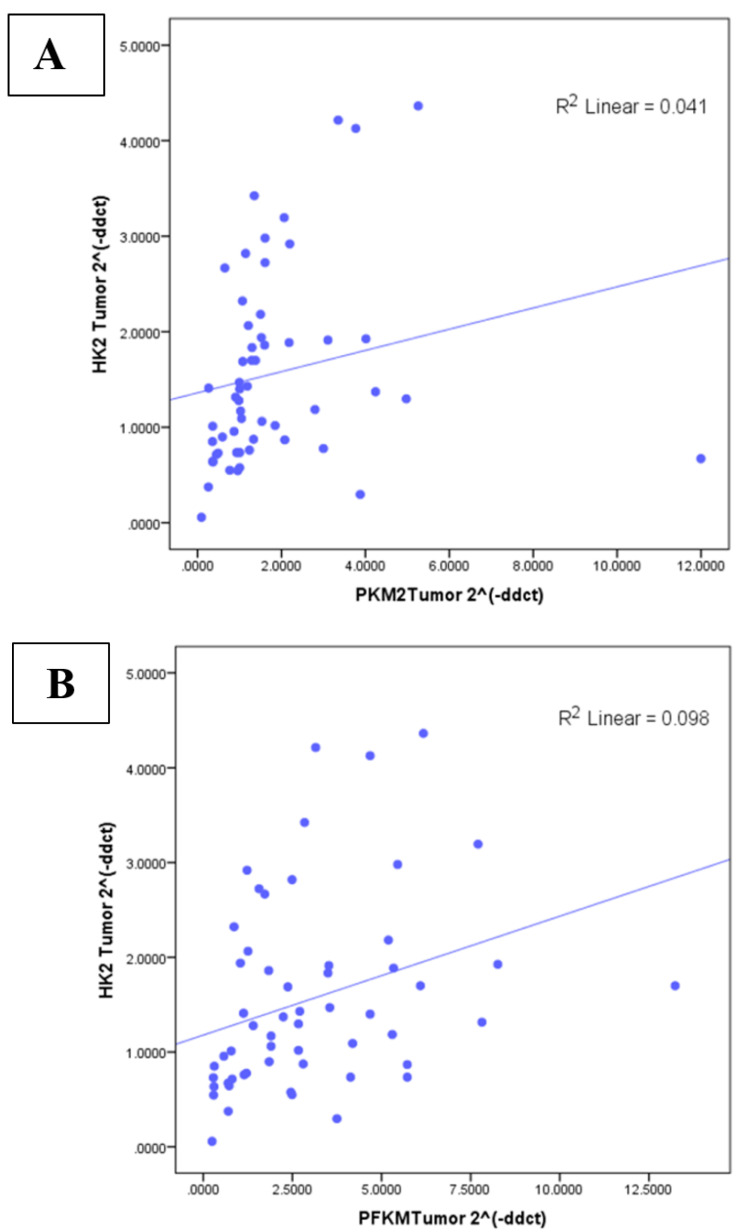

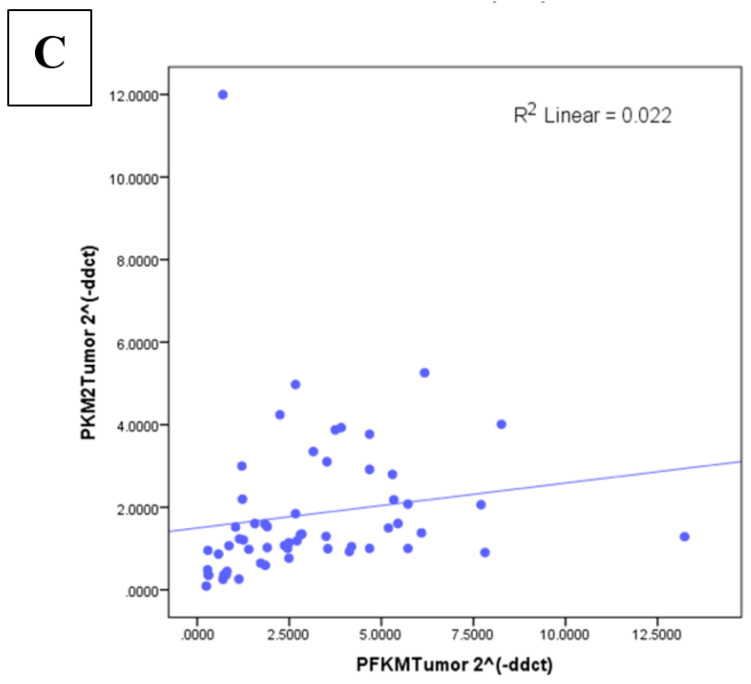
Correlation between glycolytic genes at transcript level of (**A**) *HK2* and *PKM2* (r = 0.476, *p* < 0.01); (**B**) HK2 and *PFKM* (r = 0.473, *p* < 0.01); (**C**) *PKM2* and *PFKM* (r = 0.501, *p* < 0.01) in breast cancer cohort.

*HK2* (r-value 0.529; *p* <  0.0001), *PFKM* (r = 0.509; *p* < 0.0001), and *PKM2* (r = 0.597; *p* < 0.0001) expression showed significant positive correlation with the *Ki67* proliferation marker as indicated in Supplementary Table S1. In addition, the correlation among the glycolytic genes was statistically significant and moderately positive, as indicated in Figure 6A–C.

## 4. Discussion

Cancer cells, due to their excessive proliferation rate, need considerable energy production. In contrast to non-transformed cells in normal physiological conditions, cancer cells reprogram their energy metabolism by prioritizing glycolysis over oxidative phosphorylation to fulfill their energy requirements. Additionally, it is hypothesized that the induction of aerobic glycolysis is also associated with aberrations in gene functions [26,27]. The current study was designed to assess the expression level of three key glycolytic genes, *HK2, PFKM*, and *PKM2*, along with their association with clinicopathological parameters and molecular subtypes in breast cancer cohort of Pakistan.

Our results showed highly significant overexpression of the crucial glycolytic genes (i.e., *HK2*, *PFKM,* and *PKM2*) in breast tumors as compared to their adjacent controls. These findings are consistent with previously reported *HK2* studies on cervical cancer [11] and gastric carcinoma [28]. Similarly, the oncogenic role of *HK2* has been studied in various cancer conditions, including lung [8], pancreatic [29], and colorectal [7] cancers; brain metastasis of breast cancer; and renal carcinoma [30]. *HK2* overexpression was found to be highly significant in these malignant tumors. Moreover, *PKM2* overexpression has been observed in a Chinese breast cancer cohort, as well as in pancreatic ductal adenocarcinoma using IHC [31,32]. However, not enough literature is available regarding *PFKM* gene overexpression in different solid tumors. A genome-wide association study and an in silico study analyzed the role of the *PFKM* as a novel breast cancer gene and as a potential therapeutic target for glycolysis, respectively [15,33]. Overexpression of *HK2* and *PKM2* has also been validated through IHC and TCGA data analysis. Elevated expression of all three glycolytic genes in tumor samples is suggestive of their role in breast cancer progression.

Although these metabolic genes were not correlated with age in several cancer cohorts, a statistically significant association of *HK2* [28,34] and *PFKM* gene overexpression with age was observed in our data. Nonetheless, *PKM2* transcript levels showed no significant association with age at disease onset in the Pakistani breast cancer cohort, which is in line with a previously published study [21]. Cancer patients with late disease onset showed higher expression of the glycolytic genes in comparison to patients with early disease onset in the present study cohort. This might be attributed to loss of p53 function with increasing age in elderly patients [35].

Expression of glycolytic genes was significantly higher in postmenopausal as compared to premenopausal women in the present cohort. Studies performed by Mandrup et al. suggest that there is a higher expression of hexokinase protein in abdominal adipose tissue as well as skeletal muscle tissue of postmenopausal women [36]. This may attribute to the estrogen production in adipose tissue; the key source of estrogen in post-menopausal women. Another probable reason for overexpression of the glycolytic genes might be the involvement of ER receptors, although estrogen and progesterone levels decrease with age and in postmenopausal women. Conversely, a study involving systematic analyses of clinical studies observed a two-fold increase in the expression of ER receptors in obese postmenopausal women [37]. Numerous studies have reported the role of estrogen and estrogen receptors in the regulation of glycolysis, whereby, increased expression of HK, PFK, and PK was also observed in female rat brains after estrogen treatment [38].

Among molecular subtypes of breast cancer, luminal B subtype patients showed a significant increase in *HK2* and *PKM2* expression, whereas most samples with upregulated *PFKM* fell into the luminal A subtype. This might be indicative of a potential interplay of these molecules with estrogen and/or progesterone in women with breast cancer. Usually, luminal B subtype tumors have a high recurrence rate, the worst prognosis, and long-term low treatment response [39]. The results are in line with previous findings [40]. Interestingly, *PFKM* was significantly associated with tumor differentiation. Expression of the glycolytic genes was higher in patients with poorly differentiated tumors in comparison to moderately differentiated and well-differentiated tumors in this study. Similarly, previous studies have also shown an association of the *HK2* gene with advanced tumor grades in cervical [11] and head and neck cancers [41].

In this study, statistically significant overexpression of glycolytic genes *HK2*, *PFKM,* and *PKM2* was observed in advanced cancer stages. Furthermore, transcript levels of these three glycolytic genes were observed to be higher in breast cancer patients with a greater number of lymph nodes involved, distant metastasis, and increased tumor size. Hence, breast tumors with overexpression of *HK2, PFKM*, and *PKM2* may potentially be more malignant due to enhanced aerobic glycolysis. The current findings involving *HK2* and *PKM2* are consistent with those of previous reports [9,34,42]. However, no significant association of *PFKM* with tumor size, TNM stage, or nodal metastasis has been previously reported. To the best of our knowledge, this study is the first one of its kind to report the expressional significance of *PFKM* in breast cancer development and progression. Moreover, a positive correlation between glycolytic genes (*HK2, PFKM,* and *PKM2*) and a high *Ki67* index was also observed, which is indicative of their association with cell proliferation and tumor aggressiveness. These findings emphasize the role of targeting glucose metabolism in tumorigenesis. Moderate positive correlation among the studied genes communicates the cross-talk between the glycolytic pathway genes during breast tumorigenesis in these patients.

Comprehensively, majority of the malignant tumors prioritize undergoing glycolysis to metabolize glucose. *HK2*, *PFKM*, and *PKM2* are the rate-limiting genes in the glycolytic pathway. Taken together, all these findings are indicative of the role of *HK2*, *PKM2,* and *PFKM* genes in tumor growth, proliferation, lympho-vascular invasion, and metastasis in breast cancer. Consistent with previous findings, these results also highlight the use of these genes as potential therapeutic targets for breast cancer.

## 5. Conclusions

Conclusively, collective expression of all three rate limiting glycolytic genes (*HK2, PFKM*, and *PKM2*) as novel cancer metabolic biomarkers can be beneficial for predicting disease aggressiveness and diagnosis. Moreover, targeting key glycolytic regulatory genes may serve as an attractive strategy in breast cancer diagnosis and treatment.

## Figures and Tables

**Table 1:** Primer sequences of the studied genes.

S. No.	Gene Name	Forward/Reverse Primer Seq	Product Size
1	*HK2*	GAGTTTGACCTGGATGTGGTTGC (Forward)	130 bps
		CCTCCATGTAGCAGGCATTGCT (Reverse)	
2	*PKM2*	AGGACCTGAGATCCGAACTG(Forward)	132 bps
		AGCCACAGGATGTTCTCGTC (Reverse)	
3	*PFKM*	ATGACCCATGAAGAGCACCA (Forward)	137 bps
		GCACCGGTGAAGATACCAAC (Reverse)	
4	*β-actin*	CTGAACCCCAAGGCCAAC (Forward)	108 bps
		AGAGGCGTACAGGGATAGCA (Reverse)	
5	*Ki-67*	GCCTTGGTCTCTTGGGAATAC (Forward)	123 bps
		GGAGATTAGGAGCCAGTTTGAG (Reverse)	

**Table 2:** Clinicopathological and demographic characterization of breast cancer samples along with expressions of *HK2*, *PKM2*, and *PFKM*.

Variables	Total (%)	*HK2* ^high^	*PKM2* ^high^	*PFKM* ^high^
Age of disease onset ≤45	30(50)	19	21	21
Age of disease onset >45	30(50)	22	23	27
Premenopausal status	25(42)	14	20	17
Postmenopausal status	35(58)	27	24	31
Laterality (left)	30(50)	19	21	23
Laterality (right)	30(50)	22	23	25
Grade I/II	45(75)	28	32	35
Grade III	15(25)	13	12	13
Stage I/II	41(68)	26	30	32
Stage III/IV	19(22)	15	14	16
N0/N1	49(82)	32	36	38
N2/N3	11(18)	9	8	10
No metastasis(M0)	57(95)	38	41	45
Distant metastasis(M1)	3(5)	3	3	3
T1/T2	40(67)	28	29	30
T3/T4	20(33)	13	15	18

N0/N1 = nodes involved ≤ 4; N2/N3 = nodes involved ≥ 4; T1/T2 = size of tumor is ≤5 cm; T3/T4 = size of tumor is ≥5 cm; M0 = cancer not spread to other parts of the body; M1 = cancer spread to other parts; % = percentage.

**Table 3:** Association of demographic and clinicopathological characteristics of the breast cancer cohort.

Variables	Total	*HK2*		*PKM2*		*PFKM*	
		Mean ± SEM	*p*-Value	Mean ± SEM	*p*-Value	Mean ± SEM	*p*-Value
Age of disease onset <45	30	1.197 ± 0.1129	0.0423 ^#^	2.112 ± 0.4516	0.5593 ^#^	2.393 ± 0.2688	0.0038 ^#^
Age of disease onset >45	30	1.786 ± 0.3399		1.599 ± 0.2239		4.495 ± 0.6518	
Premenopausal status	25	1.011 ± 0.1066	0.0233 ^#^	0.9701 ± 0.1295	0.0192 ^#^	2.229 ± 0.3476	0.0183 ^#^
Postmenopausal status	35	1.912 ± 0.3129		1.741 ± 0.2095		4.073 ± 0.5835	
Laterality (left)	30	1.553 ± 0.3226	0.1257 ^#^	1.044 ± 0.1162	0.0488 ^#^	2.584 ± 0.3395	0.5137 ^#^
Laterality (right)	30	1.909 ± 0.3522		2.233 ± 0.4911		3.368 ± 0.5807	
Tumor	60	1.948 ± 0.2848	<0.0001 ^¥^	1.960 ± 0.09343	<0.0001 ^¥^	3.056 ± 0.3269	<0.0001 ^¥^
Control	60	1.000 ± 0.1055		1.000 ± 0.2483		1.000 ± 0.15330	
Grade I/II	45	1.058 ± 0.05400	0.1121 ^#^	1.309 ± 0.1353	0.2237 ^#^	1.976 ± 0.2425	0.0403 ^#^
Grade III	15	1.314 ± 0.1560		2.165 ± 0.6173		2.987 ± 0.4276	
Stage I/II	41	1.496 ± 0.1572	0.0459 ^#^	1.161 ± 0.1164	0.0302 ^#^	2.786 ± 0.4199	0.0459 ^#^
Stage III/IV	19	2.694 ± 0.6708		1.881 ± 0.2609		1.241 ± 0.1621	
N0/N1	49	1.595 ± 0.2565	0.0010 ^#^	1.582 ± 0.1863	0.0084 ^#^	2.123 ± 0.2261	0.0078 ^#^
N2/N3	11	3.171 ± 0.5819		3.529 ± 0.8762		4.985 ± 1.071	
No metastasis (M0)	57	2.259 ± 0.6133	0.0039 ^#^	1.620 ± 0.1726	0.0088 ^#^	2.015 ± 0.2073	0.0432 ^#^
Distant metastasis (M1)	3	7.009 ± 1.794		4.066 ± 0.6310		4.816 ± 1.835	
T Stage: T1/T2	40	1.349 ± 0.1343	0.0499 ^#^	1.652 ± 0.2400	0.0090 ^#^	1.801 ± 0.2317	0.0026 ^#^
T Stage: T3/T4	20	2.299 ± 0.5588		2.480 ± 0.4631		3.205 ± 0.3991	

^¥^ Wilcoxon signed rank test; ^#^ Mann–Whitney U test; SEM = standard error mean.

## Data Availability

Available upon request from the corresponding author.

## Supplementary Materials

The following supporting information can be downloaded at: https://www.mdpi.com/article/10.3390/genes13030549/s1, Figure S1: Expression of glycolytic markers in molecular subtypes of breast cancer (tumor vs. paired control). Fold change of glycolytic gene in (A) HK2; (B) PFKM; (C) PKM2. Significance level * *p* < 0.05, ** *p* < 0.001 *** *p* < 0.0001.; Figure S2: Overall survival analysis using Log rank (Mantel-Cox) test of HK2 (HR = 1.951; *p* = 0.1), PFKM (HR = 2.03; *p* = 0.1) and PKM2 (HR = 0.6; *p* = 0.4) genes in breast cancer cohort. Table S1: Correlation of HK2, PFKM and PKM2 gene with Ki-67 (proliferation marker). * Spearmen correlation, all bold values are significant having *p* < 0.05.

## Abbreviations

OXPHOS	Oxidative phosphorylation
HK	Hexokinase
PFK	Phosphofructokinase
PK	Pyruvate kinase
G-6P	Glucose-6-phosphate
*PFKM*	Phosphofructokinase muscle
*PKM2*	Pyruvate kinase isozyme M2
*HK2*	Hexokinase2
IHC	Immunohistochemistry
qPCR	Quantitative polymerase chain reaction
*Ki67*	Marker of proliferation
HIF1	Hypoxia inducible factor
cDNA	Complimentary DNA
mRNA	Messenger RNA
bps	Base pairs
TCGA	The Cancer Genome Atlas
TNBC	Triple-negative breast cancer

## References

[1] Tseng P.L., Chen C.W., Hu K.H., Cheng H.C., Lin Y.H., Tsai W.H., Cheng T.J., Wu W.H., Yeh C.W., Lin C.C. (2018). The decrease of glycolytic enzyme hexokinase 1 accelerates tumor malignancy via deregulating energy metabolism but sensitizes cancer cells to 2-deoxyglucose inhibition. Oncotarget.

[2] Salvador M.M., de Cedrón M.G., Rubio J.M., Martínez S.F., Martínez R.S., Casado E., de Molina A.R., Sereno M. (2017). Lipid metabolism and lung cancer. Crit. Rev. Oncol./Hematol..

[3] Kalyanaraman B. (2017). Teaching the basics of cancer metabolism: Developing antitumor strategies by exploiting the differences between normal and cancer cell metabolism. Redox Biol..

[4] Fadaka A., Ajiboye B., Ojo O., Adewale O., Olayide I., Emuowhochere R. (2017). Biology of glucose metabolization in cancer cells. J. Oncol. Sci..

[5] Wu Z., Wu J., Zhao Q., Fu S., Jin J. (2020). Emerging roles of aerobic glycolysis in breast cancer. Clin. Transl. Oncol..

[6] Perrin-Cocon L., Vidalain P.-O., Jacquemin C., Aublin-Gex A., Olmstead K., Panthu B., Rautureau G.J.P., André P., Nyczka P., Hütt M.-T. (2021). A hexokinase isoenzyme switch in human liver cancer cells promotes lipogenesis and enhances innate immunity. Commun. Biol..

[7] Hamabe A., Yamamoto H., Konno M., Uemura M., Nishimura J., Hata T., Takemasa I., Mizushima T., Nishida N., Kawamoto K. (2014). Combined evaluation of hexokinase 2 and phosphorylated pyruvate dehydrogenase-E1α in invasive front lesions of colorectal tumors predicts cancer metabolism and patient prognosis. Cancer Sci..

[8] Zhao Y., Li N., Zhao J., Shi S. (2020). High expression of hexokinase 2 promotes lung cancer proliferation and metastasis. Arch. Med. Sci..

[9] Wu J., Hu L., Hu F., Zou L., He T. (2017). Poor prognosis of hexokinase 2 overexpression in solid tumors of digestive system: A meta-analysis. Oncotarget.

[10] Sun Z., Tan Z., Peng C., Yi W. (2021). HK2 is associated with the Warburg effect and proliferation in liver cancer: Targets for effective therapy with glycyrrhizin Corrigendum in/10.3892/mmr. 2021.12143. Mol. Med. Rep..

[11] Liu C., Wang X., Zhang Y. (2019). The roles of HK2 on tumorigenesis of cervical cancer. Technol. Cancer Res. Treat..

[12] Ausina P., Da Silva D., Majerowicz D., Zancan P., Sola-Penna M. (2018). Insulin specifically regulates expression of liver and muscle phosphofructokinase isoforms. Biomed. Pharmacother..

[13] Li X.B., Gu J.D., Zhou Q.H. (2015). Review of aerobic glycolysis and its key enzymes–new targets for lung cancer therapy. Thoracic cancer.

[14] Imbert-Fernandez Y., Clem B.F., O’Neal J., Kerr D.A., Spaulding R., Lanceta L., Clem A.L., Telang S., Chesney J. (2014). Estradiol stimulates glucose metabolism via 6-phosphofructo-2-kinase (PFKFB3). J. Biol. Chem..

[15] Rani Y., Kaur K., Sharma M., Kalia N. (2020). In silico analysis of SNPs in human phosphofructokinase, Muscle (PFKM) gene: An apparent therapeutic target of aerobic glycolysis and cancer. Gene Rep..

[16] Li X., Kim W., Arif M., Gao C., Hober A., Kotol D., Strandberg L., Forsström B., Åsa S., Oksvold P. (2021). Discovery of functional alternatively spliced PKM transcripts in human cancers. Cancers.

[17] Walls J.F., Subleski J.J., Palmieri E.M., Gonzalez-Cotto M., Gardiner C.M., McVicar D.W., Finlay D.K. (2020). Metabolic but not transcriptional regulation by PKM2 is important for natural killer cell responses. Elife.

[18] Hitosugi T., Kang S., Heiden M.G.V., Chung T.-W., Elf S., Lythgoe K., Dong S., Lonial S., Wang X., Chen G.Z. (2009). Tyrosine phosphorylation inhibits PKM2 to promote the Warburg effect and tumor growth. Sci. Signal..

[19] Desai S., Ding M., Wang B., Lu Z., Zhao Q., Shaw K., Yung W.A., Weinstein J.N., Tan M., Yao J. (2014). Tissue-specific isoform switch and DNA hypomethylation of the pyruvate kinase PKM gene in human cancers. Oncotarget.

[20] Chen X., Chen S., Yu D. (2020). Protein kinase function of pyruvate kinase M2 and cancer. Cancer Cell Int..

[21] Zhang X., He C., He C., Chen B., Liu Y., Kong M., Wang C., Lin L., Dong Y., Sheng H. (2013). Nuclear PKM2 expression predicts poor prognosis in patients with esophageal squamous cell carcinoma. Pathol. Res. Pract..

[22] Marbaniang C., Kma L. (2018). Dysregulation of glucose metabolism by oncogenes and tumor suppressors in cancer cells. Asian Pac. J. Cancer Prev. APJCP.

[23] Yan X., Hu Y., Wang B., Wang S., Zhang X. (2020). Metabolic dysregulation contributes to the progression of Alzheimer’s disease. Front. Neurosci..

[24] Riaz S.K., Khan J.S., Shah S.T.A., Wang F., Ye L., Jiang W.G., Malik M.F.A. (2018). Involvement of hedgehog pathway in early onset, aggressive molecular subtypes and metastatic potential of breast cancer. Cell Commun. Signal..

[25] Livak K.J., Schmittgen T.D. (2001). Analysis of relative gene expression data using real-time quantitative PCR and the 2^−ΔΔCT^ method. Methods.

[26] Yu L., Chen X., Sun X., Wang L., Chen S. (2017). The glycolytic switch in tumors: How many players are involved?. J. Cancer.

[27] Epstein T., Gatenby R.A., Brown J.S. (2017). The Warburg effect as an adaptation of cancer cells to rapid fluctuations in energy demand. PLoS ONE.

[28] Rho M., Kim J., Jee C.D., Lee Y.M., Lee H.E., Kim M.A., Lee H.S., Kim W.H. (2007). Expression of type 2 hexokinase and mito chondria-related genes in gastric carcinoma tissues and cell lines. Anticancer Res..

[29] Anderson M., Marayati R., Moffitt R., Yeh J.J. (2017). Hexokinase 2 promotes tumor growth and metastasis by regulating lactate production in pancreatic cancer. Oncotarget.

[30] Liu H., Liu N., Cheng Y., Jin W., Zhang P., Wang X., Yang H., Xu X., Wang Z., Tu Y. (2017). Hexokinase 2 (HK2), the tumor promoter in glioma, is downregulated by miR-218/Bmi1 pathway. PLoS ONE.

[31] Lockney N., Zhang M., Lu Y., Sopha S.C., Washington M.K., Merchant N.B., Zhao Z., Shyr Y., Chakravarthy A.B., Xia F. (2015). Pyruvate kinase muscle isoenzyme 2 (PKM2) expression is associated with overall survival in pancreatic ductal adenocarcinoma. J. Gastrointest. Cancer.

[32] Xiao H., Zhang L., Chen Y., Zhou C., Wang X., Wang D., Liu Z. (2020). PKM2 Promotes Breast Cancer Progression by Regulating Epithelial Mesenchymal Transition. Anal. Cell. Pathol..

[33] Ahsan H., Halpern J., Kibriya M.G., Pierce B., Tong L., Gamazon E., McGuire V., Felberg A., Shi J., Jasmine F. (2014). A genome-wide association study of early-onset breast cancer identifies PFKM as a novel breast cancer gene and supports a common genetic spectrum for breast cancer at any age. Cancer Epidemiol. Prev. Biomark..

[34] Ogawa H., Nagano H., Konno M., Eguchi H., Koseki J., Kawamoto K., Nishida N., Colvin H., Tomokuni A., Tomimaru Y. (2015). The combination of the expression of hexokinase 2 and pyruvate kinase M2 is a prognostic marker in patients with pancreatic cancer. Mol. Clin. Oncol..

[35] Salminen A., Kaarniranta K. (2010). Glycolysis links p53 function with NF-κB signaling: Impact on cancer and aging process. J. Cell. Physiol..

[36] Mandrup C.M., Roland C.B., Egelund J., Nyberg M., Enevoldsen L.H., Kjaer A., Clemmensen A., Christensen A.N., Suetta C., Frikke-Schmidt R. (2020). Effects of High-Intensity Exercise Training on Adipose Tissue Mass, Glucose Uptake and Protein Content in Pre-and Post-menopausal Women. Front. Sports Act. Living.

[37] Boonyaratanakornkit V., Pateetin P. (2015). The role of ovarian sex steroids in metabolic homeostasis, obesity, and postmenopausal breast cancer: Molecular mechanisms and therapeutic implications. BioMed Res. Int..

[38] Chen J.-Q., Brown T.R., Russo J. (2009). Regulation of energy metabolism pathways by estrogens and estrogenic chemicals and potential implications in obesity associated with increased exposure to endocrine disruptors. Biochim. Biophys. Acta (BBA)-Mol. Cell Res..

[39] Serrano-Carbajal E.A., Espinal-Enriquez J., Hernández-Lemus E. (2020). Targeting metabolic deregulation landscapes in breast cancer subtypes. Front. Oncol..

[40] Martínez-Ordoñez A., Seoane S., Avila L., Eiro N., Macía M., Arias E., Pereira F., García-Caballero T., Gómez-Lado N., Aguiar P. (2021). POU1F1 transcription factor induces metabolic reprogramming and breast cancer progression via LDHA regulation. Oncogene.

[41] Li W.C., Huang C.H., Hsieh Y.T., Chen T.Y., Cheng L.H., Chen C.Y., Liu C.J., Chen H.M., Huang C.L., Lo J.F. (2020). Regulatory Role of Hexokinase 2 in Modulating Head and Neck Tumorigenesis. Front. Oncol..

[42] Li H., Yan M., Wu X., Wang Y., Huang L. (2021). Expression and clinical significance of pyruvate kinase M2 in breast cancer: A protocol for meta-analysis and bioinformatics validation analysis. Medicine.

