# Supplementary material for: Expression of HK2, PKM2, and PFKM Is Associated with Metastasis and Late Disease Onset in Breast Cancer Patients
Source: Genes (Basel). 2022 Mar 20;13(3):549. doi: 10.3390/genes13030549 (PMC8955648; doi:10.3390/genes13030549)
Supplement: Supplementary file 1 [file genes-13-00549-s001.zip › genes-1605831-supplementary.pdf]

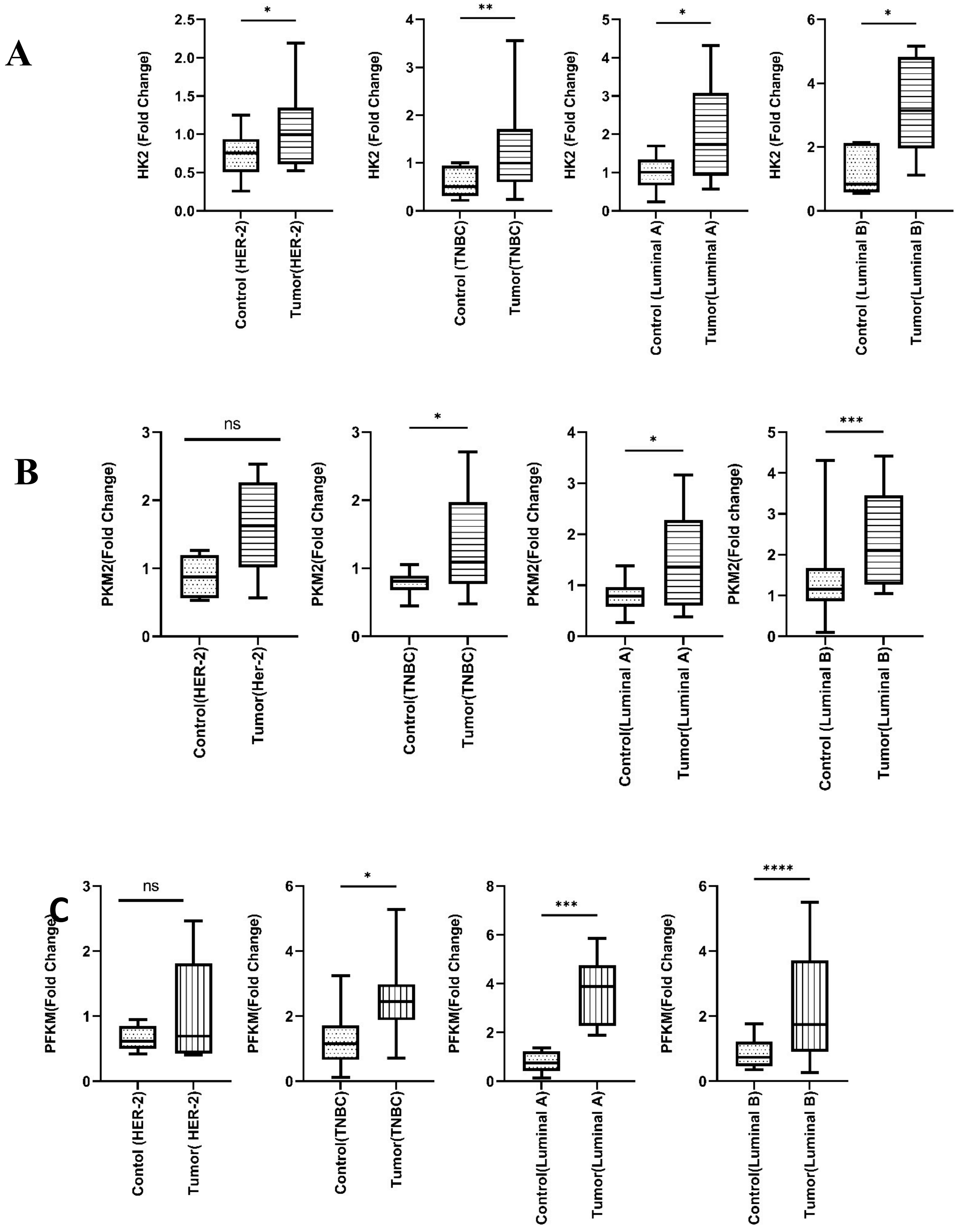

Supplementary Figure S1: Expression of glycolytic markers in molecular subtypes of breast cancer (tumor vs paired control). Fold change of glycolytic gene in A) *HK2*; B) *PFKM*; C) *PKM2*. Significance level *p<0.05, **p<0.001 ***p<0.0001.

Supplementary Table S1: Correlation of *HK2, PFKM* and *PKM2* gene with *Ki-67* (proliferation marker). *Spearmen correlation, all bold values are significant having p<0.05

	Ki-67	Hk2	PFKM	PKM2
**Ki-67(r-value)**	1	**0.529**	**0.509**	**0.597**
**p-value**		<0.0001	<0.0001	<0.0001

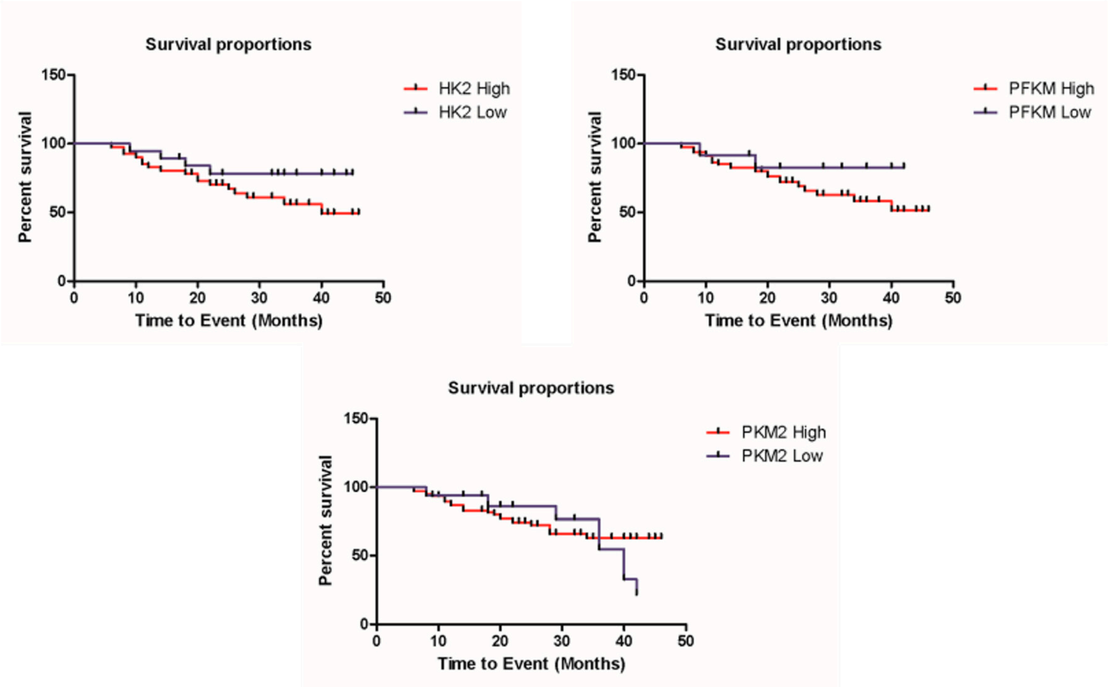

Supplementary Figure S2: Overall survival analysis using Log rank (Mantel-Cox) test of *HK2* (HR=1.951; p=0.1), *PFKM* (HR=2.03; p=0.1) and *PKM2* (HR=0.6; p=0.4) genes in breast cancer cohort.
